# YAP Expression is Related to the Aggressive Behavior of Odontogenic Cysts

**DOI:** 10.1055/s-0044-1801275

**Published:** 2025-05-01

**Authors:** Jutamas Uttagomol, Adjabhak Wongviriya, Ariya Chantaramanee, Thanit Prasitsak

**Affiliations:** 1 Department of Oral Diagnosis, Faculty of Dentistry, Naresuan University, Phitsanulok, Thailand; 2 Department of Preventive Dentistry, Faculty of Dentistry, Naresuan University, Phitsanulok, Thailand; 3 Department of Oral Biology, Faculty of Dentistry, Naresuan University, Phitsanulok, Thailand

**Keywords:** odontogenic cysts, odontogenic keratocysts, YAP, Ki-67, Yes-associated protein, Immunohistochemistry

## Abstract

**Objective:**

Yes-associated protein (YAP) and Ki-67 are known to be involved in cell proliferation. While their overexpression is associated with aggressive behaviors in oral squamous cell carcinoma (OSCC), their roles in odontogenic cysts have not been thoroughly investigated. Therefore, this study aimed to evaluate the immunohistochemical expression of YAP and Ki-67 in odontogenic cysts.

**Material and Methods:**

Tissue samples included 12 radicular cysts, 10 dentigerous cysts, 9 odontogenic keratocysts, and 9 normal oral mucosa specimens. Immunohistochemical analysis was conducted both manually and using ImageJ software for comparison.

**Statistical analysis:**

The Kruskal–Wallis, followed by the Mann–Whitney *U* -test was used to assess the differences in the YAP and Ki-67 immunoexpression levels among various group lesions. The correlation between those two proteins in individual groups was tested by Spearman correlation. A *p* -value of less than 0.05 was considered statistically significant. SPSS software version 25.0 was used.

**Results:**

There was a strong nuclear staining of YAP in basal and superficial cells and an intense cytoplasmic YAP positivity in odontogenic keratocysts, whereas a weaker YAP staining in both the nucleus and cytoplasm throughout the epithelial thickness was observed in dentigerous cyst, radicular cyst, and normal oral mucosa, respectively. YAP expression differed significantly in all odontogenic cysts compared with normal oral mucosa ( *p* < 0.05). Ki-67 expression was notably higher in odontogenic keratocysts relative to other cysts and normal oral mucosa. Although no statistically significant correlation was found between YAP and Ki-67 across the groups, both proteins displayed similar positive trends in odontogenic keratocysts.

**Conclusion:**

These findings suggest that YAP activation may be related to the proliferative behavior of odontogenic cysts, especially in more aggressive lesions, but less likely to influence the inflammatory cysts. This insight could improve understanding of their pathogenesis and pave the way for new diagnostic and therapeutic approaches.

## Introduction

Yes-associated protein (YAP) is an important effector involved in the transduction of signals in the Hippo pathway. 1 This protein functions as a transcriptional coactivator that shuttles between the cell nucleus and cytoplasm, and its location affects its function. Upon activation, YAP shifts from the cell cytoplasm to the nucleus and binds to the promoters of target genes by interacting with TEAD to regulate genes involved in cell proliferation, migration, differentiation, and cell death. 2 3 4 5 During the normal wound healing process, nuclear YAP accumulation increases in the basal layer of skin to maintain cell proliferation of progenitor cells in normal skin development and to promote wound healing. 6 Studies have revealed that YAP hyperactivation plays an essential role in the rapid progression, tumorigenic phenotypes, metastasis, and poor prognosis of oral squamous cell carcinoma (OSCC). 7 8 YAP has been studied extensively in various cancers. Recently, more attention has focused on YAP in craniofacial diseases and hard tissue remodeling 9 including odontogenic tumors. 10 11 Nonetheless, its role in odontogenic cysts remains limited.

Odontogenic cysts are defined as bony cavities within the jaws lined with epithelium. They are subclassified as developmental cysts or inflammatory cysts in origin. 12 The epithelium of odontogenic cysts is originally derived from the reduced enamel epithelium, remnants of Malassez, dental lamina remnants, degenerated enamel organ, or the surface epithelium of mucosa. 13 The frequently occurring odontogenic cysts in the oral cavity are the radicular cyst (RC), dentigerous cyst (DC), and odontogenic keratocyst (OKC). 14 RC arises as a result of inflammation, whereas DC and OKC are developmental cysts. 12 Even though odontogenic cysts typically exhibit a gradual and expansive growth pattern, certain types, such as OKC, show more aggressive behavior. 15 A higher level of a cell proliferation marker, Ki-67, is evident in OKC compared with the inflammatory cysts and the other developmental cysts. 16 17 These findings indicate that OKC demonstrates a different growth mechanism from RC and DC. However, the underlying mechanisms are still not fully understood. It is noteworthy to investigate YAP expression in odontogenic cysts, as this could enhance the understanding of the role of this molecule in disease pathogenesis. Therefore, the current study aimed to evaluate the immunohistochemical expression of YAP protein in normal oral mucosa (NOM), RC, DC, and OKC, and to assess its correlation with Ki-67, a proliferation marker.

## Materials and Methods

### Specimens

This retrospective study included all tissue specimens of odontogenic lesions collected at the Oral Diagnostic Pathology Unit, Oral Diagnosis Department, Faculty of Dentistry, Naresuan University, Phitsanulok, Thailand. The study was approved by the Naresuan University Institutional Review Board (COE No. 139/2022).

Following a review of histologic findings, a total of 41 odontogenic lesions were selected. These included RCs ( *n* = 12), DCs ( *n* = 10), OKCs ( *n* = 9), and NOM ( *n* = 9). Only specimens with adequate odontogenic epithelium were included. OKCs associated with Gorlin syndrome and specimens with extensive inflammatory components, with the exception of RCs, were excluded from the study.

### Immunohistochemistry

Immunohistochemical staining was performed on 4-µm thick paraffin-embedded sections mounted on positively charged microscope slides (Epredia Superfrost Plus Adhesion, Thermo Fisher Scientific, MA, United States). After dewaxing and rehydration, antigen retrieval was achieved by heating the sections at 90–95°C in citrate buffer (ab93678, Abcam, Cambridge, UK) with a pH of 6 for 20 min. Endogenous peroxidase activity was blocked by immersing the sections in BLOXALL Blocking Solution (Vector, CA, United States) for 10 min at room temperature, followed by three washes in phosphate-buffered saline with 0.2% Tween 20 for 5 min each. The sections were then treated with normal goat serum for 60 min at room temperature. Primary antibodies against YAP (D8H1X, Cell Signaling Technology, MA, United States; 1:200, 4°C overnight) and Ki-67 (ab15580, Abcam, Cambridge, UK; 1:500, 4°C overnight) were applied. Immunoreaction was developed using the Vectastain Elite ABC-HRP Kit (Vector, CA, United States) with diaminobenzidine (Sigmafast 3,3′-diaminobenzidine tablets, Sigma-Aldrich, MA, USA) as the chromogen for 5 min. The sections were then counterstained with Mayer's hematoxylin and mounted. Tissue sections of OSCC served as the positive control, and sections with the primary antibody omitted were used as negative controls. Then, immunohistochemistry images of each sample were visualized and captured using a microscope slide scanner (Zeiss, Germany) at 20X objective lens (1 pixels = 0.5 μm).

### Immunohistochemical Assessment

Endogenous YAP expression was evaluated using a semiquantitative method adapted from Anand et al 10 and Remmele and Stenger. 18 Points were assigned based on (a) the percentage of cells with positive nuclear or cytoplasmic staining: 0 = 0%, 1 = 1 to 25%, 2 = 26 to 50%, 3 = 51 to 75%, and 4 = > 75%, and (b) the intensity of the immunoreaction, scored from 0 to 3 points. The final result, termed the Immunoreactive Score (IRS), was calculated by multiplying these two parameters, resulting in a score range of 0 to 12 points.

For Ki-67, scoring was performed by counting the percentage of positive cells in five high-power representative fields, with the following score: 0 = 0%, 1 = 1 to 25%, 2 = 26 to 50%, and 3 = > 50%, as adapted from Alsaegh et al. 19

### Image Analysis

**Fig. 1:**
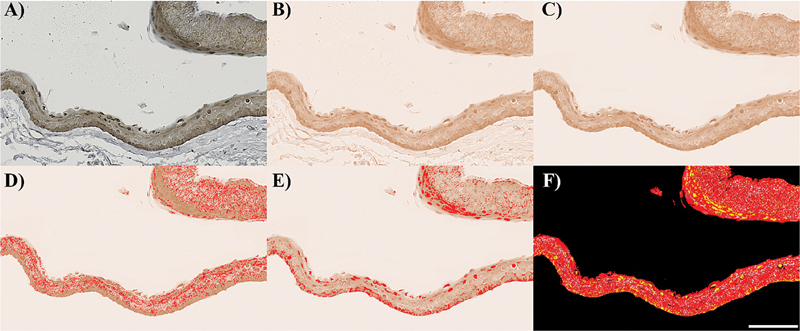
The determination of the area fraction of YAP1-positive staining within the nucleus (example of odontogenic keratocyst). (A) Immunohistochemistry image. (B) The original image was deconvoluted; only the DAB-stained image was selected. (C) Connective tissue was removed. (D) Positive DAB staining within the cytoplasm was segmented. (E) Positive DAB staining within the nucleus was segmented. (F) The merged image shows the area with positive DAB staining within the cytoplasm (white) and the nucleus (yellow) compared with the whole epithelial area (red). (20 × objective lens; 1 pixel = 0.5 μm; scale bar represents 100 μm). DAB, 3,3′-diaminobenzidine; YAP, Yes-associated protein.

To analyze YAP expression in the nucleus and cytoplasm, immunohistochemistry images of each sample were obtained using a microscope slide scanner (Zeiss, Germany). Images were analyzed using ImageJ software 20 ( Fig. 1A ). Deconvolution was performed using the Deconvolution2 plugin 21 ( Fig. 1B ). Only 3,3′-diaminobenzidine (DAB) stained slides were used for further analysis. The connective tissue areas were removed ( Fig. 1C ). The epithelium and nuclei were manually segmented and measured ( Fig. 1D–E ). Any gaps within the segmented epithelial image were filled. Since each type of cyst varies in area, the study determined stain positivity using the area fraction ( Fig. 1F ). The area fraction of YAP-positive staining within the cytoplasm was calculated using the following formula:

Area fraction of YAP-positive cytoplasm = Positive area in cytoplasm × 100

Total epithelial area

Similarly, the area fraction of YAP-positive staining in the nucleus was calculated using:

Area fraction of YAP-positive nucleus = Positive area in nucleus × 100

Total epithelial area

### Statistical Analysis

The data were analyzed using SPSS software version 25.0 (IBM Corp., Armonk, NY, United States). Descriptive data, including demographic information and area fraction, were presented as percentages and means ± SD, while immunohistochemical scores were presented as median: first quartile (Q1) and third quartile (Q3). Differences in the expression levels of YAP and Ki-67 among various types of odontogenic lesions and NOM were assessed using the Kruskal–Wallis test, followed by Mann–Whitney *U* -test. To evaluate the correlation between the expression levels of YAP and Ki-67 within each group (NOM, RC, DC, and OKC), Spearman's rank correlation coefficient was calculated. A *p* -value of less than 0.05 was considered statistically significant.

## Results

### Demographic and Clinical Data

The demographic parameters are summarized in Table 1 . The sample exhibited a predominance of males (67.75%) compared with females (32.25%). Patient ages ranged from 14 to 60 years, with a mean age of 35.17 ± 14.42 years. The peak incidence occurred between 21 and 30 years of age. There was a slight predilection for the maxilla (61.29%) over the mandible (38.71%), with the anterior region being the most commonly affected site (51.61%). The most frequent radiographic feature observed was unilocular radiolucency (87.10%).

**Table 1:** Demographic characteristics of patients

Parameters	RC *n* (%)	DC *n* (%)	OKC *n* (%)	Total *n* (%)
Gender				
Male	10 (83.3)	8 (80)	3 (33.33)	21 (67.75)
Female	2 (16.7)	2 (20)	6 (66.67)	10 (32.25)
Age				
10–20	1 (8.33)	2 (20)	2 (22.22)	5 (16.14)
21–30	2 (16.67)	5 (50)	3 (33.33)	10 (32.25)
31–40	1 (8.33)	1 (10)	1 (11.11)	3 (9.67)
41–50	5 (41.67)	1 (10)	1 (11.11)	7 (22.58)
51–60	2 (16.67)	1 (10)	2 (22.22)	5 (16.13)
NS	1 (8.33)	0 (0)	0 (0)	1 (3.23)
Anatomical site				
Maxilla	7 (58.33)	8 (80)	4 (44.44)	19 (61.29)
Mandible	5 (41.67)	2 (20)	5 (55.56)	12 (38.71)
Region				
Anterior	7 (58.34)	8 (80)	1 (77.78)	16 (51.61)
Posterior	4 (33.33)	2 (20)	7 (11.11)	13 (41.93)
Anterior + posterior	1 (8.33)	0 (0)	1 (11.11)	2 (6.46)
Radiographic features				
Unilocular	10 (83.3)	10 (100)	7 (77.78)	27 (87.10)
Multilocular	1 (8.33)	0 (0)	1 (11.11)	2 (6.45)
NS	1 (8.33)	0 (0)	1 (11.11)	2 (6.45)

Abbreviations: DC, dentigerous cyst; NS, not state; OKC, odontogenic keratocyst; RC, radicular cyst.

### YAP Expression

**Fig. 2:**
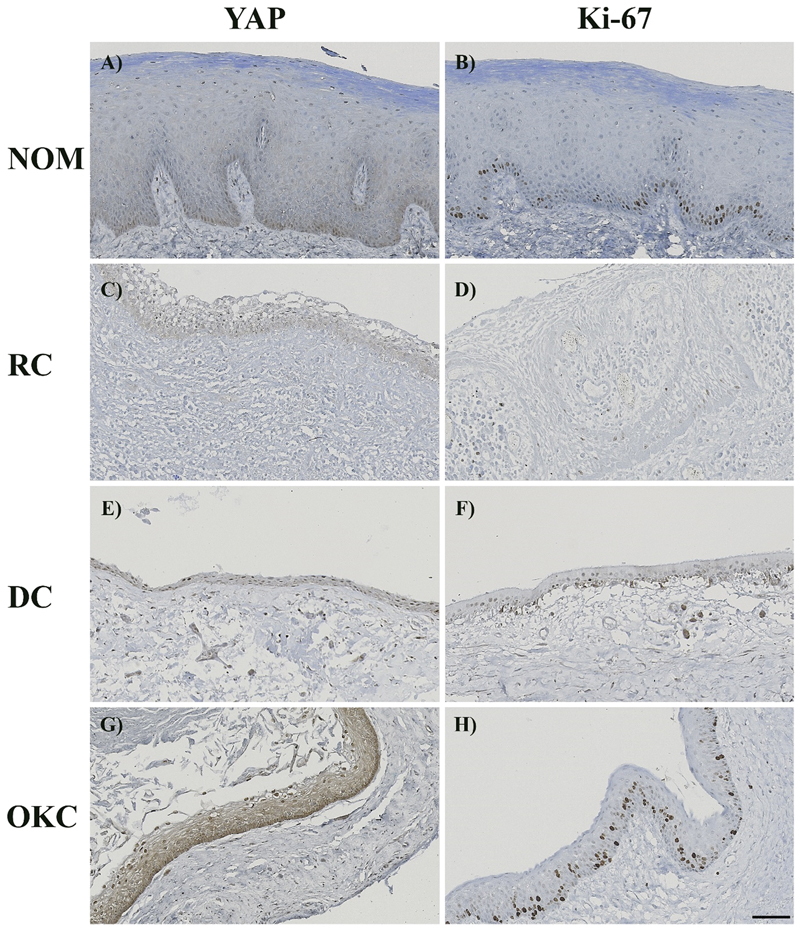
Immunoexpression of YAP and Ki-67 in odontogenic cysts and NOM. (A) YAP expression in NOM. (B) Ki-67 expression in NOM. (C) YAP expression in RC. (D) Ki-67 expression in RC. (E) YAP expression in DC. (F) Ki-67 expression in DC. (G) YAP expression in OKC. (H) Ki-67 expression in OKC. OKC exhibited stronger YAP positivity throughout the entire epithelium, while NOM, RC, and DC showed YAP positivity predominantly in the basal layer. Ki-67 is strongly expressed in both the suprabasal and basal layers of OKC, whereas NOM, RC, and DC displayed weaker Ki-67 expression limited to the basal layer. (Scale bar represents 100 μm.) DC, dentigerous cyst; NOM, normal oral mucosa; OKC, odontogenic keratocyst; RC, radicular cyst; YAP, Yes-associated protein.

**Fig. 3:**
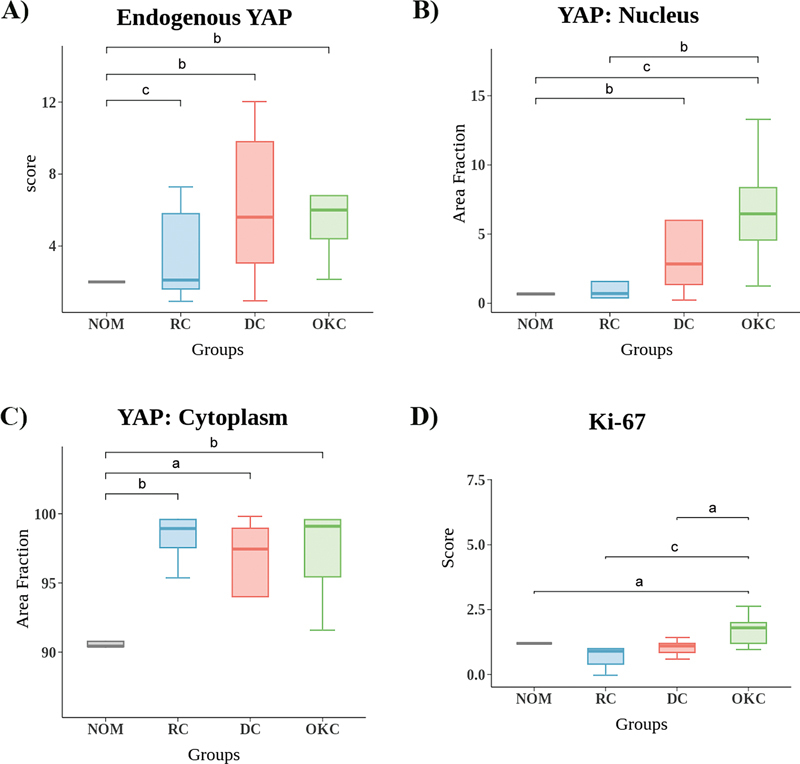
The comparison of endogenous YAP, nuclear YAP, cytoplasmic YAP, and Ki-67 expression between odontogenic cysts and NOM. (A) Box plot showing the endogenous YAP expression scores across different groups (NOM, RC, DC, OKC). (B) Box plot demonstrating the area fraction of YAP expression in the nucleus for each group. (C) Box plot demonstrating the area fraction of YAP expression in the cytoplasm for each group. (D) Box plot representing the Ki-67 expression scores across the different groups. (Kruskal–Wallis test followed by pairwise comparisons with the Mann–Whitney *U* -test; ^a^ *p* < 0.05; ^b^ *p* < 0.01; ^c^ *p* < 0.001). DC, dentigerous cyst; NOM, normal oral mucosa; OKC, odontogenic keratocyst; RC, radicular cyst; YAP, Yes-associated protein.

Both normal epithelium and odontogenic cysts expressed YAP but in different intensities and patterns ( Fig. 2 ). The highest level of endogenous YAP-positive staining was observed in OKC (6.00: 4.40, 6.80). YAP staining was distributed strongly in the nucleus of OKC's basal and superficial epithelial layers as well as in the cell cytoplasm of the whole epithelial thickness ( Fig. 2G ). RC (2.10: 1.60, 5.80) and DC (5.60: 3.05, 9.80) shared similar results as weak YAP immunostaining was noted in the cell nucleus and cytoplasm throughout the entire thickness ( Fig. 2C , 2E ). In NOM (2.00: 2.00, 2.00), YAP distribution predominantly in the basal cell nuclei was seen with the lowest expression level in the cell cytoplasm ( Fig. 2A ). All odontogenic cysts revealed a significant difference in endogenous YAP expression compared with NOM ( *p* < 0.05) ( Fig. 3A ).

To confirm the results, the study further evaluated the area fraction of YAP positivity within the nucleus and cytoplasm using ImageJ. Consistent with the previous findings, OKC exhibited a high expression of YAP in the nucleus (6.46 ± 4.47), followed by DC (2.83 ± 5.04), RC (0.70 ± 3.70), and NOM (0.67 ± 0.20) ( Fig. 3B ). Concerning the YAP-positive area in the cytoplasm, all cysts showed significantly higher YAP immunoreactivity compared with NOM ( *p* < 0.05), although there was no statistically significant difference in YAP protein levels among the individual cysts ( Fig. 3C ).

### Ki-67 Expression

OKC (1.90: 1.20, 2.00) revealed the highest expression level of Ki-67 among all groups with a predominantly presence at the suprabasal and basal cell layers ( Fig. 2H , 3D ). The distribution of Ki-67 protein was observed mainly at the basal epithelial layer in DC (1.10: 0.85, 1.20) and NOM (1.20: 1.20, 1.20) which showed slightly stronger intensity in the latter. Ki-67 expression was randomly found at the basal cell layers in RC (0.90: 0.40, 1.00) ( Fig. 2B , 2D , 2F , 3D ).

### Correlation between Immunostaining of YAP and Ki-67

The correlation between endogenous YAP and the proliferative marker, Ki-67 was determined in individual cysts and NOM ( Table 2 ). The result demonstrated no statistically significant correlation between the two proteins in all groups. However, YAP and Ki-67 expression in OKC revealed the same positive trend.

**Table 2:** Spearman correlation coefficient ( *r* ) and statistical significance ( *p* ) for the correlation between YAP and Ki-67 between different groups

Group correlation	*r* (Spearman)	*p* (Spearman)
**Normal oral mucosa (NOM)** **YAP** × **Ki-67**	0.124	0.751
**Radicular cyst (RC)** **YAP** × **Ki-67**	0.019	0.953
**Dentigerous cyst (DC)** **YAP** × **Ki-67**	0.074	0.838
**Odontogenic** k **eratocyst (OKC)** **YAP** × **Ki-67**	0.359	0.343

Abbreviation: YAP, Yes-associated protein.

## Discussion

YAP is an important downstream effector of the Hippo pathway. Its activity is negatively inhibited when the Hippo pathway is activated through its kinase cascade, resulting in phosphorylation of YAP and subsequent sequestration of YAP in the cell cytoplasm, either by retention or degradation. 22 23 Once the Hippo pathway is inactive, unphosphorylated YAP translocates to the cell nucleus and regulates genes involved in cell proliferation, migration, cell growth, apoptosis, and tissue homeostasis. 2 3 24 Therefore, the YAP shuttle between the cell nucleus and cytoplasm is not static and affects its functions. 25 YAP has been reported to act as an oncogene in various cancers such as ovarian cancer, hepatocellular carcinoma, nonsmall cell lung carcinoma, gastric cancer, 26 27 28 29 and oral squamous cell carcinoma. In oral squamous cell carcinoma, YAP relates to the initiation and progression of tumor 7 30 and correlates with poor prognosis. 8 While most research has focused on YAP immunoexpression in cancers or premalignant conditions, its expression in odontogenic cysts is limited.

The result of the present study demonstrates a significantly elevated YAP and Ki-67 immunoreactivity in OKC compared with RC, DC, and NOM. These findings highlight a pronounced expression of YAP in OKC and reinforce the association between YAP and cellular proliferation in this cyst type. This finding was consistent with prior studies. 31 32 Man and colleagues studied the expression levels of YAP/TAZ and downstream proteins in NOM and OKC. It was reported that a significant upregulation of YAP, in the basal and superficial epithelial cell nuclei, and its downstream proteins (Cyr61, CTGF) was detected in OKC compared with NOM. Additionally, OKC also demonstrated significantly elevated mRNA levels of transcription factors (TEAD1, TEAD4, RUNX2) and cell cycle-related genes (CDK2, PCNA). Moreover, Ki-67 protein was increased in the nucleus of the basal cell layer and demonstrated a synchronous distribution in double-labeling immunofluorescence with YAP/TAZ in OKC samples. 31 Similar results were shown in another recent study by Gonçalo et al. The highest immunoreactivity level of YAP was mainly in the basal and suprabasal cells in OKC followed by ameloblastoma, DC, and dental follicle (DF). There was a positive correlation between YAP and Ki-67 in OKC and a statistically significant correlation between YAP and Ki-67 in unicystic ameloblastoma. 32 The highly expressed YAP in OKC may be due to its shared characteristics of both cysts and tumors.

In terms of cyst characteristics, the present study revealed that both inflammatory and developmental cysts expressed YAP. Compared with NOM, a significantly elevated immunoreactivity of endogenous and cytoplasmic YAP was detected in all cysts. However, regarding the nuclear fraction of YAP, OKC exhibited the highest levels of YAP, followed by DC, RC, and NOM, respectively. This distribution closely mirrored the pattern observed for Ki-67, a proliferative marker. These findings, in accordance with previous reports, 31 32 suggested that YAP may be associated with the high growth potential and aggressive behavior of developmental cysts, especially the more aggressive lesions, by enhancing proliferation activity. As RC and DC expanded through fluid accumulation, 33 the proliferation of their epithelial lining cells occurred as a secondary response to cystic enlargement. Conversely, the primary growth mechanism of OKC was attributed to the high proliferative activity of its epithelial lining. 34 35

Regarding tumor-like properties, OKC has a different growth behavior from the other odontogenic cysts and normal tissue 36 and the epithelial lining has a high proliferative potential. 17 Highly expressed YAP protein in OKC's cell nucleus and cytoplasm in this current study reflected its high proliferative potential and neoplastic behavior, and, along with previous studies, 31 32 collectively underscored the plausible association between YAP and the proliferative activity in OKC. A previous study on precancerous lesions and OSCC has reported that the degree of YAP expression varies depending on tissue type and cancer severity. 37 In NOM and epithelial hyperplasia, YAP is weakly expressed in both the nucleus and cytoplasm. In contrast, high-grade oral epithelial dysplasia (OED), carcinoma in situ (CIS), and OSCC exhibit strong YAP expression in both cellular compartments. This suggests a correlation between elevated YAP expression and increased malignancy. 37 However, in the present study, no statistically significant correlation between YAP and Ki-67 was observed, although their expression demonstrated a similar positive trend in OKC. This may be attributed to the possibility that the activation status of YAP is associated with the distinct growth potential of OKC.

Regarding the location of positive staining, the results of the present study show that YAP was positively stained in both the nucleus and cytoplasm, with the highest level of YAP staining observed in the nucleus of OKC among all groups followed by DC, RC, and NOM, respectively. All cysts exhibited a notable increase in cytoplasmic YAP positivity compared with NOM. However, varying intensities of cytoplasmic YAP staining were observed across the cystic lesions. RC and DC displayed low levels of cytoplasmic YAP, while OKC exhibited intense cytoplasmic YAP labeling. This finding indicates not only the intensity, but also the pattern of YAP expression may reflect tissue characteristics and functions. A study in esophageal and gastric tissue reported an increased nuclear localization of YAP indicates enhanced proliferation, maintains an undifferentiated state 38 and acts as an oncogene involved in intestinal transformation via WNT signaling, which is crucial for the maintenance and expansion of undifferentiated progenitor cells. On the contrary, cytoplasmic YAP is essential for epithelial progenitor cell differentiation in adult airways 39 and functions as a tumor suppressor by suppressing the WNT/β-catenin signaling pathway. 40 In normal human skin, YAP nuclear localization is noted in basal layer progenitor cells, while other differentiating cells feature cytoplasmic YAP. Some nuclear YAP expression is observed in highly flattened squamous cells that have differentiated. Additionally, YAP accumulation in the nucleus is elevated to maintain the proliferation of basal cell progenitor cells and to promote healing after wounding. 6 Integrin-Src and EGFR-PI3K signaling are proposed to regulate YAP nuclear localization in keratinocytes. The differentiated daughter cells in the epithelium lose contact with the basement membrane extracellular matrix and lack integrin-Src and EGFR-PI3K signaling, resulting in less accumulation of YAP in the nucleus. The immunoreactivity of YAP in the cell nucleus and cytoplasm appears to relate to dysplasia and malignant phenotype. This abundant expression of YAP may arise from gene amplification, transcription, or protein stability. 37 38

A limitation of this study is the absence of nuclear staining in the image analysis, which resulted in an inability to determine the total number of cells. Consequently, the percentage of YAP-positive cells relative to the total cell population could not be determined. In addition, further investigations involving a wider variety of odontogenic cyst types and a larger sample size may provide deeper insights into the role of YAP in the progression of odontogenic cysts. Improved understanding of disease pathogenesis could contribute to the development of targeted therapeutics, especially for more aggressive odontogenic cysts.

## Conclusion

Activated YAP may influence the proliferative behavior of the epithelial lining in odontogenic cysts, especially in more aggressive cases, but less likely to affect the inflammatory cysts. This finding could enhance knowledge of their pathogenesis and lead to the development of novel diagnostic and therapeutic strategies.
